# The extension of the largest generalized-eigenvalue based distance metric *D_ij_(γ*_1_) in arbitrary feature spaces to classify composite data points

**DOI:** 10.5808/GI.2019.17.4.e39

**Published:** 2019-11-14

**Authors:** Mosaab Daoud

**Affiliations:** Independent Research Scientist, Toronto, ON M1S1G2, Canada

**Keywords:** classiﬁcation, clustering, composite data points, limiting dispersion map, linear (non-linear) transformation function, sets of sequences, statistical learning

## Abstract

Analyzing patterns in data points embedded in linear and non-linear feature spaces is considered as one of the common research problems among different research areas, for example: data mining, machine learning, pattern recognition, and multivariate analysis. In this paper, data points are heterogeneous sets of biosequences (composite data points). A composite data point is a set of ordinary data points (e.g., set of feature vectors). We theoretically extend the derivation of the largest generalized eigenvalue-based distance metric *D_ij_* (*γ*_1_) in any linear and non-linear feature spaces. We prove that *D_ij_* (*γ*_1_) is a metric under any linear and non-linear feature transformation function. We show the sufficiency and efficiency of using the decision rule δΞi (i.e., mean of *D_ij_* (*γ*_1_)) in classification of heterogeneous sets of biosequences compared with the decision rules *min*_Ξ__i_ and *median*_Ξ__i_. We analyze the impact of linear and non-linear transformation functions on classifying/clustering collections of heterogeneous sets of biosequences. The impact of the length of a sequence in a heterogeneous sequence-set generated by simulation on the classification and clustering results in linear and non-linear feature spaces is empirically shown in this paper. We propose a new concept: the limiting dispersion map of the existing clusters in heterogeneous sets of biosequences embedded in linear and nonlinear feature spaces, which is based on the limiting distribution of nucleotide compositions estimated from real data sets. Finally, the empirical conclusions and the scientific evidences are deduced from the experiments to support the theoretical side stated in this paper.

## Introduction

Biological databases are the normal hosts for bio-sequences. Analyzing bio-sequences is the main role of the sequence analysis research field. Biological databases are organized based on either (1) information and knowledge that is implicitly associated with bio-sequences, or (2) information and knowledge that is extracted from bio-sequences. In this paper, the key words bio-sequences and sequences have the same meaning, and we use them interchangeably. The process of submitting sequences by the existing scientific research labs is a continuous process. Therefore, the volumes of the existing biological databases are increasing continuously. On the other hand, to capture and analyze the useful and undetectable information contained in biological datasets, the sequence analysis research community is encouraged to propose the next-generation of sequence analysis methods, algorithms and techniques. It should be noted that the existing sequence analysis methods, algorithms and techniques are categorized into different research fields, for example: machine learning, pattern recognition, data mining, bioinformatics, and signal processing.

Plants, organisms and microorganisms are classified into different classes. In the 19th century, Francis Galton [1], the first geneticist, studied data collected from different types of peas. He studied measurements (i.e., features) extracted from parent and offspring. Classification is a natural human process, which can be performed by the human brain to classify different types of entities. Computationally speaking, the classification process can be performed by computational devices (i.e., machines) using a well-defined classification algorithm. The classification process can be performed using two different modes: either (1) classification or (2) clustering. The data availability and the availability of information about data are considered key points in selecting the appropriate mode to perform the required classification process. The classification process can be performed using: (1) statistical approaches, (2) artificial neural network (ANN) approaches, and (3) syntactical approaches [2,3]. The existing approaches can only process ordinary data points (e.g., feature vectors or sequences). An ordinary data point is a one-entity data point, for example: observation of feature variable (uni-variate), feature vector, string or sequence.

As we mentioned, in this paper, the data points under consideration are sets of heterogeneous sequences (composite data points). A composite data point is a multi-entity data point, for example: set of feature vectors, set of strings or sequences. The extracted information from sets of heterogeneous sequences (composite data points) can be evaluated as either perfect or imperfect information [4]. The imperfect information has an impact on the decision-making process. Hence, there are two types of decisions: (1) risky decisions (type-1) and (2) decisions with uncertainty (type-2) [4]. The risky decision [4] is a decision with the following attribute: its risk can be analyzed or interpreted by a probabilistic model or a fuzzy model. The uncertainty decision [4] is a decision with the following attribute: its risk cannot be analyzed or interpreted by a probabilistic model or a fuzzy model. The risk is defined as the gray area between certainty and uncertainty. If the sequence-based datasets under consideration are collected to represent a biological phenomenon (e.g., viral infection, spread of diseases), then type-2 decisions are considered unsafe. Part of the solution lies in drawing up a road map for developing the next-generation of feature extraction, and sequence-analysis techniques. To pave the path for the researchers in the field, in this paper, we aim to tackle the problem of analyzing sequence-sets from a different angle. The generalized largest eigenvalue-based distance metric *D_ij_* (*γ*_1_) proposed in Daoud’s study [5] (defined in Daoud’s study [6] as Mosaab-metric Space) can be discovered in a different way. We aim to extend the theoretical and practical sides of *D_ij_* (*γ*_1_) in any linear and nonlinear feature spaces. Moreover, we use the key words: group of sequences, sequence-set, and set of sequences interchangeably.

The remaining sections of this paper are summarized as follows. In the next part of this section we present the related work. Section I presents the extension of the largest generalized eigenvalue-based distance metric in any linear/non-linear feature spaces. Section II presents the experiments and results. Finally, conclusions and future work are given in section III.

In genetics, the datasets under consideration are sequence-based datasets, where each data point is either a biological sequence (ordinary data point) or a set of biological sequences (composite data point). There are three types of biological sequences. The types of biological sequences are defined as follows: (1) DNA sequences, (2) RNA sequences, and (3) PROTEIN sequences. In terms of language modeling, each type of biological sequences is drawn from a different alphabet. The alphabets of DNA, RNA, and PROTEIN sequences are defined as follows: Σ_*DNA*_={A, C, G, T}, Σ_*RNA*_={A, C, G, U}, and Σ_*PROTEIN*_={A, R, N, D, C, Q, E, G, H, I, L, K, M, F, P, S, T, W, Y, V} respectively. The sequences are either classified or clustered (grouped) based on their biological features (e.g., homology). For example, in nature, the segmented genome of influenza virus is a homology-free group of sequences. In fact, the segmented genome of influenza virus is considered as a heterogeneous sequence-set, because its sequences have different biological functions and different nucleotide compositions. Moreover, there is another type of sequence-set, the homogeneous sequence-set, where sequences are grouped in a sequence-set based on their common biological features (e.g., sharing a common ancestor) using various algorithms, for example, multiple alignment algorithms, pairwise alignment algorithms, and alignment-free algorithms.

In the recent years, the capacity of the research work in the area of sequence analysis has been developed rapidly and extensively, and the objective is to analyze different types of sequences at different molecular levels (e.g., primary structure, secondary structure). Analyzing sequence-sets in feature spaces is a new developing research direction. Daoud and Kremer established a new platform for the new research direction: *Alignment-free Sequence-Set Analysis* [5-7], and achieved the first successful attempt in 2010. The new research direction basically focuses on analyzing patterns in classes of sequence-sets without using alignment. In the next part of this section, we present the related research work.

**Fig. 1.**
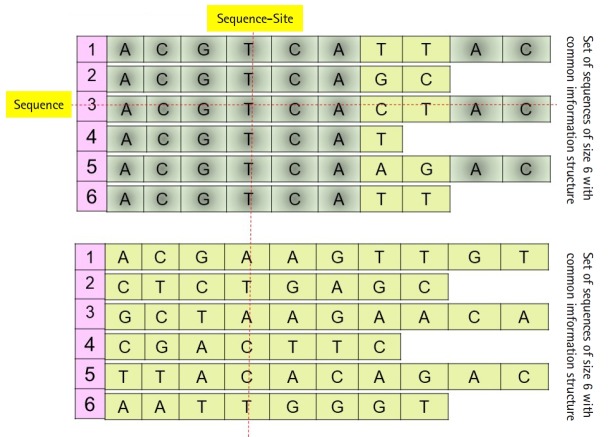
Example: set of sequences with or without common information structure.

Daoud and Kremer [8] proposed a new technique to extract feature vectors embedded in R^*p*^ from sets of homogeneous sequences (e.g., families of biological sequences), to implement statistical and neural classification techniques on homogeneous sequence-sets in linear feature space using the linear transformation *X=(X_1_, X_2_, …, X_p_)'*, instead of data space. The proposed technique works vertically on the sequences of each independently aligned homogeneous sequence-set. Precisely, instead of mapping each sequence of a homogeneous sequences-set into the feature space (i.e., each sequence is represented by one observed feature vector or data vector *χ*), the technique map the whole homogeneous sequences-set into linear feature space by converting each ordered pair of sequence-set sites into data vector in order to preserve the common hidden information structure in homogeneous sequences-sets (Fig. 1), unaligned sequence-set with common information structure). Moreover, Daoud and Kremer [8] proposed a new classification algorithm to classify aligned homogeneous sequences-sets in linear feature space. The proposed statistical classification algorithm is considered as a variance-covariance structure-based classification algorithm [8], where the optimization on the statistical side is defined in terms of statistical variation to capture biological variation in homogeneous sequence-sets. Hence, the proposed theory connects the statistical variation as a statistical concept with the biological variation as a biological concept. The classification algorithm is built upon using the following largest generalized eigenvalue-based distance metric:

(1) $$D_{\mathrm{ij}}(\gamma_{1})=|\gamma_{1}^{'}(\Omega_{i}-\Omega_{j})\gamma_{1}|=|\lambda_{1}|>0,$$

where *γ_1_* the largest generalized eigenvector associated with *λ*_1_, the largest generalized eigenvalue of the matrix (Ω_i_-Ω_j_), and Ω_i_ and Ω_j_ are the variance-covariance matrices of the sequence-sets *i* and *j* respectively. *D_ij_* (*γ*_1_) is a (matrix inverse operation)-free distance metric. In addition, Daoud [5] solved the sequence-set proximity problem under the homology-free assumption, which is defined as the problem of measuring the closeness between any two sets of bio-sequences (two composite data points), where the homology assumption is unknown within each sequence-set or between sequence-sets. It is a generalization of the sequence proximity problem. The sequence proximity problem is defined as the problem of measuring the distance between any two given sequences, or among the sequences of a given sequence-set in a pairwise manner. It should be noted that the existing (1) pairwise alignment, (2) multiple alignment, and (3) alignment-free based distance/similarity measures are designed to solve the sequence proximity problem under the homology assumption [9,10]. The generalization of sequence proximity problem shrinks the effectiveness and the validity of the existing alignment-based and alignment-free distance/similarity measures, thus, a distance measure at the sequence-set level is required [5] to perform the following tasks on sequence-sets under the homology-free assumption: (1) searching, (2) classification, and (3) clustering, (4) detecting variation, and (5) visualization. The proposed distance metric given in 1 shows robustness in performing the required tasks on sequence-sets under the homology-free assumption. The time complexity of the proposed distance metric is linear while the time complexity of local alignment-based distance measures is quadratic [5,6]. Comparisons between the proposed largest generalized eigenvalue-based distance metric *D_ij_* (*γ*_1_) and the alignment-based distance measures are given in Daoud’s study [1,3], and the results show robustness in terms of selectivity, sensitivity, and time complexity. Moreover, the proposed algorithms in Daoud et al’s study [5,6,8,11] are designed using the following principles.

(*P*_0_) Homogeneous sequence-set must be mapped from data space into feature space as one entity to preserve its hidden common information structures. It is expected that the sequences of any homogeneous sequence-set have common information structures. In feature space, a statistical assumption-free representation is considered, for example, the variance-covariance structure, and a variance-covariance structure-based distance measure is proposed to design supervised and unsupervised distance-based classifiers (Fig. 1).

(*P*_1_) Heterogeneous sequence-set must be mapped from data space into feature space as separated sub-entities (i.e., as separated sequences), since there is no prior knowledge about the existence of common information structures among those sub-entities. In feature space, statistical assumption-free representation is considered, which is the variance-covariance structure, and a variance-covariance structure-based distance measure is proposed to design supervised and unsupervised distance-based classifiers (Fig. 1).

The common corner in both principles is the variance-covariance structure. The variance-covariance structure is a statistical information structure with the following characteristic: it is a relation descriptor which can be used to statistically describe all possible relations between feature variables of a feature vector embedded in R*^p^* in terms of co-variation and variation. It has a matrix form, which it is embedded in R*^p^* × R*^p^*. To solve the sequence-set proximity problem under the homology-free assumption, a variance-covariance structure-based distance measure (or metric) is required to achieve this goal. The most popular variance-covariance structure-based distance measures is the Mahalanobis distance measure. The computation of Mahalanobis distance measure requires the inverse of the variance-covariance matrix. Hence, the measure is inapplicable in the case of singular matrices, in addition, the matrix inverse operation is computationally expensive operation. The singularity of variance-covariance matrices shrinks the applicability of well know multivariate statistical analysis techniques, for example: principal components analysis (PCA), factor analysis, variance-covariance matrices-based test statistics, unless a new matrix transformation is defined. In this case and in terms of time complexity, more computations will be added.

Forstner metric is a mathematical metric that can be used in measuring the difference between two variance-covariance matrices [12]. It is entirely based on only the generalized eigen-problem of two variance-covariance matrices. The metric is defined as the sum of squared logarithms of the eigenvalues of $\left(\sum_{1}^{-1}\sum_{2.}\right)$. Therefore, Forstner metric requires the inverse of one of the variance-covariance matrices. Hence, the metric is inapplicable in case of singular matrices, in addition, computationally, the matrix inverse operation is an expensive operation. The metric has no statistical interpretation.

**Fig. 2.**
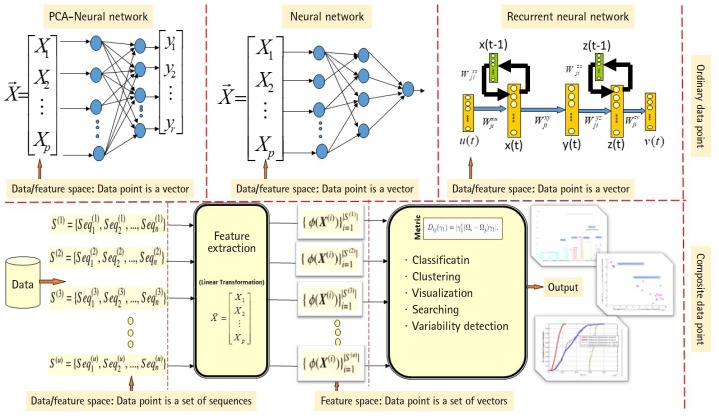
The comparison between the existing machine learning approaches and the proposed variance-covariance statistical pattern recognition system.

ANN are well known stochastic approximation models and powerful in performing classification tasks. The Vanilla back-propagation ANN used in Daoud and Kremer’s study [8] to classify aligned homogeneous sequence-sets (aligned RNA families) in linear feature space. The network trained with the standard gradient descent approach implemented by the generalized delta rule. The proposed ANN-based algorithm shows its effectiveness in classifying aligned homogeneous sequences-sets (aligned RNA families) in linear feature space [8]. Moreover, Daoud and Kremer [11] proposed a novel algorithm for detecting similarities between aligned homogeneous sequence-sets in linear feature space using the steady state concept of PCA-neural network. The proposed algorithm designed using the valuable equilibrium property of the PCA–neural network, which is defined as: training the PCA–neural network with two sets of feature vectors using the generalized Hebbian rule, where each set of feature vectors represents an aligned homogeneous sequence-set, may lead the PCA-neural network to converge to the same attractor point or to two different attractor points. In this context, the attractor point is defined in terms of the principal axises (i.e., eigenvector). ANN and PCA–neural network can only process ordinary data points, therefore, we implemented computational modification to process composite data points. As a conclusion, the computational modification shrinks the capability of those stochastic approximation models to process large number of composite data points. For example, at each computational phase, we can compare two composite data points using the steady state concept of PCA–neural network to conclude similarities or dissimilarities. As we mentioned, one of the effectiveness of the new variance-covariance structure-based statistical pattern recognition system proposed in Daoud’s study [5] is its capability to process large number of composite data points, specifically, heterogeneous sets of sequences. The comparison between the existing machine learning approaches and the proposed variance-covariance statistical pattern recognition system is given in Fig. 2.

The statistical variation is a well-known measure (statistic) in statistical sciences, and it is rapidly used in life sciences to measure and analyze biological variation in biological datasets. The generalized form of the statistical variation is the variance-covariance structure, which is represented by the variance-covariance matrix. The variance-covariance matrix is a symmetric positive definite matrix that represents a summary of variations and co-variations of a vector of feature variables. The off-diagonal elements of the variance-covariance matrix are the co-variances of feature variables, while the diagonal elements of the variance-covariance matrix represent the variances of feature variables [1]. The distribution of the eigenvalues of variance-covariance matrices has been studied in multivariate statistical analysis [13]. The problem of comparing two variance-covariance matrices has been studied extensively in the areas of multivariate statistical analysis and applied statistics, and it is reduced to the problem of analyzing the generalized eigenstructure (i.e., eigenvectors associated with eigenvalues) of two or more variance-covariance matrices. The existing generalized models of PCA are as follows: (1) the Generalized Principal Component Analysis Model (GPCA) [14], (2) the Common Principal Components Analysis Model (CPCA) [15,16], and (3) the MD-Generalized Principal Component Analysis Model (MD-GPCA) [17] are entirely different from the concepts of (a) distance measure, (b) generalized distance measure, (c) metric and metric space, (d) generalized metric and generalized metric space. Those models are defined in terms of the generalized eigenstructure (i.e., eigenvectors associated with eigenvalues) of well-defined functions of variance-covariance matrices $\left(e.g.,\;f\left(\sum_{1},\sum_{2}\right)=\left(\sum_{1}^{-1}\sum_{2}\right)\right)$. All those theoretical models are statistical assumption-based models. The input of Generalized Models of PCA are feature vectors (ordinary data points), and the outputs are generalized eigenvectors and generalized eigenvalues (i.e., they are not distance values). At each computational phase, the Generalized Principal Component Analysis Models (GPCA and MD-GPCA) can process two composite data points. The Common GPCA can process few composite data points under the following statistical assumptions: feature vectors are assumed to have multivariate normal distributions, and covariance matrices must be non-singular matrices. Therefore, we note that, both Forstner metric and Mahalanobis distance measure are different from GPCAs proposed in Flury and colleagues’ studies [14-17]. At this point, Comparisons between the proposed metric *D_ij_* (*γ*_1_) and the existing PCA-based (dis)-similarity comparison models GPCAs can be found in Daoud’s study [5], which are approximately identical to the above comparisons. In other words, the concept of *D_ij_* (*γ*_1_) is different from the concepts of all the existing GPCAs.

In the case of heterogeneous sequence-sets, the mixture model is the appropriate statistical model that can be applied in analyzing heterogeneous sequence-sets in feature space. Learning from a mixture model is not an easy task due to the characteristics of the model. For example, the model can only process ordinary data points (observed feature vectors/data vectors). In addition, defining a weighted probability distribution function for the mixture model requires a statistical estimation technique to estimate its weights (core parameters). As a matter of fact, the estimation technique and the data availability have an impact on the decision-making process. In this context, we can shape the following: most of the proposed statistical techniques are derived using statistical assumptions. Those assumptions can be easily violated due to the nature of real-world problems. The violation of statistical assumptions may jeopardize the performance of the existing statistical techniques in analyzing datasets under consideration. Consequently, the next-generation of statistical learning models are expected to be assumption-free models, and hence, they can be implemented on a wide range of datasets. For example, if the statistical decision rule is derived using the following statistical assumption: the feature vector has a specific probability distribution (e.g., multivariate normal distribution), then it is not necessary that the assumed feature vector follows the same probability distribution in all datasets under consideration. The assumption is expected to face violation in real-world problems, and different datasets are expected to have different probability distributions.

**Fig. 3.**
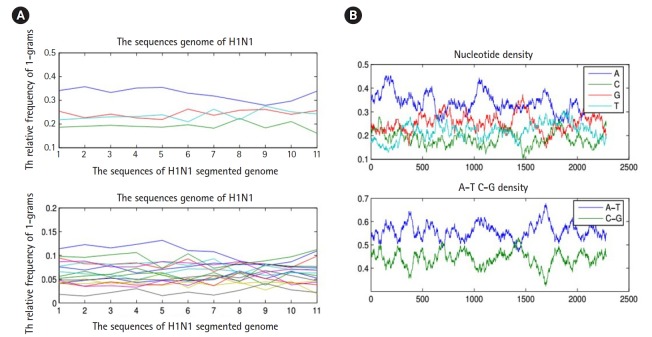
(A) The nucleotide density of each biological sequence in a segmented genome of a influenza virus (a composite data point) using 1-grams and 2-grams feature vectors. (B) Figures counts the number of each type of base or word in a biological sequence using matlab-bioinformatics toolbox.

To proceed further in presenting the research work, we must present the following facts. Any biological sequence is linear in time. Statistically speaking, any biological sequence is defined as ordered symbols (i.e., bases or nucleotides), where those symbols are drawn from a finite alphabet based on a specific probability distribution (i.e., nucleotide composition). This statistical assumption is not always true, and by performing the following simple experiment, we can confirm this fact. By sliding a window on any biological sequence from one end to another, the resultant local probability distributions of the nucleotide composition are not always homogeneous (i.e., having same probability distribution along any biological sequence) (Fig. 3). Thus, in the case of a homogeneous sequence-set, it is not always biological sequences that constitute a sequence-set have homogeneous probability distribution, but they are homogeneous in the sense of sharing the same ancestor. Thus, those sequences are biologically homogeneous, but statistically, the nucleotide composition of each sequence is hard to be modeled by one probability distribution. This fact is true for any heterogeneous sequence-set, and thus it is a violation for the statistical assumption of the mixture model (i.e., probability distributions are specified in advance), and in this case, the mixture model is inapplicable.

In this paper, we anticipate that the proposed metric D_ij_ (γ_1_) in Daoud’s studies [5,6] can be theoretically and practically extended in any linear and nonlinear feature spaces to solve the sequence-set proximity problem under the homology-free assumption. In this section, we presented the related work, in the next subsection, the statement of the problem is presented.

### The statement of the problem (the extension of D_ij_ (γ_1_) in any linear and nonlinear feature spaces)

Our work is exclusively focuses on the following: we aim to extend the theoretical-side and practical-side of the metric D_ij_ (γ_1_) in any linear and nonlinear feature spaces, where each data point is a set of heterogeneous sequences (i.e., each data point is a composite data point or a dataset). We aim to show the efficiency and sufficiency of using the mean of the distance values of D_ij_ (γ_1_) instead of using the minimum or the median of the distance values in solving the classification problem of heterogeneous sequence-sets in any linear and nonlinear feature spaces. We aim to analyze the impact of linear and non-linear transformation functions on classifying/clustering collections of heterogeneous sets of biosequences. We aim to show the impact of the sequence length on the classification and clustering of simulated heterogeneous sequence-sets generated form real heterogeneous sequence-sets in linear and nonlinear feature spaces.

It should be noted that all the existing data mining and machine learning methods are ordinary data point-based methods (e.g., observed feature vector, sequence). Generalization from ordinary data point to composite data point (e.g., set of observed feature-vectors, set of sequences) has not been achieved yet by the research communities in the fields of data mining and machine learning. Transforming data points from a one feature space to another linear or nonlinear feature space has the effect of detecting varieties of undetectable (dis)-similarities among data points.

After we presented the statement of the research problem under consideration, the objectives of this paper are entirely different from the objectives of the research work presented in Daoud et al’s studies [5,6,8,11,18]. However, the objectives of this paper are considered as core objectives of the research topic: Alignment-free Sequence-set Analysis or implicitly Bio-Data Mining of Composite Data Points. Moreover, the research work presented in this paper is an extension of the research work presented in Daoud et al’s studies [5,6,8,11,18]. In the next section, the extension of *D_ij_* (*γ*_1_) in any linear and nonlinear feature spaces is presented.

## Methods

### The extension of *D_ij_* (*γ*_1_) in any linear and nonlinear feature spaces

In this section, we present the theoretical extension of *D_ij_* (*γ*_1_) in any linear and nonlinear feature spaces. *D_ij_* (*γ*_1_) [5,6,18] is a variance-covariance structure based distance measure, that can measure the distance between any two variance-covariance matrices embedded in (R*^p^* × R*^p^*). The measure is a matrix inverse operation-free measure which can work with singular matrices and it requires less computation compared with Mahalanobis distance measure and Forstner metric. The measure is built upon the generalized GPCA, and implicitly the generalized eigen-problem, but conceptually it is irrelevant to the concept of a model. In fact, it is a measure. *D_ij_* (*γ*_1_) requires no prior statistical-based assumptions, which make it easy to implement, and thus, it is an assumption-free distance measure. The datasets under consideration are sequence-based datasets. Sequences may vary in length, and in terms of uncertainty, we assume that each sequence is generated by a stochastic source, in other words, ∃ a statistical model (Model) such that the nucleotide composition of a given sequence can be modeled using (Model). If sequences in a sequence-set have the same biological function and implicitly have the same nucleotide composition, then it is called a homogeneous sequence-set, otherwise, it is called a heterogeneous sequence-set. Usually, each data point embedded in the data space is a sequence. Suppose that each data point embedded in the data space is a heterogeneous set of sequences. In this context, and for each heterogeneous sequence-set embedded in data space, it is hard to assume unrealistic assumptions, for example: the nucleotide composition of each sequence can be modeled by one probability distribution or by probability distributions that are generalizable to all other sequences in a heterogeneous sequence-set.

The stages of data life cycle have an impact on the data mining phase and decision-making phase. In any dataset, the existence of hidden information structures is expected, therefore, it is required to map datasets under consideration into various feature spaces to recognize, analyze, and visualize the existence of hidden information structures. This strategy has an impact on decision making phase. As we mentioned, sequence-sets are embedded in data space, which can be projected into feature spaces. Hence, there are various data mining methods that can be used to analyze datasets embedded in data space and feature spaces. The new paradigm shift proposed by Daoud [5] is constructed upon the following new concept: we have to map any sequence-based dataset into various feature spaces in order to recognize, analyze, and visualize its hidden information structures from different angles using suitable data mining methods. In other words, the new paradigm shift has one principle, which is the extension principle of data projection. We define the extension principle of data projection as: we have to extend the data life cycle by mapping datasets into various feature spaces, and consequently, we have to extend adaptability and applicability of methods used in analyzing datasets. In the next part of this section, we present the theoretical extension of *D_ij_* (*γ*_1_) in any linear and nonlinear feature spaces.

#### Definition 1

Let *Class of Spaces* = {*Sp*_1_, *Sp*_2_, *Sp*_3_, …} be the set of all possible feature spaces into which data points can be projected. Let *ϕ* be a well-defined transformation function that can be used to map data points from one feature space to another. Thus, using the extension principle of data projection, the data life cycle always can be extended by finding a transformation function *ϕ* that can be used to map data points from one feature space to another.

#### Definition 2

Let Σ = {*σ_1_,σ_2_,…,σ_r_*} be a finite alphabet. Without loss of generality, let $S^{\left(j\right)}=\left\{Seq_{1}^{\left(j\right)},Seq_{2}^{\left(j\right)},\ldots ,Seq_{n}^{\left(j\right)}\right\}$ be a set of sequences, where $Seq_{i}^{\left(j\right)}$ is the *i^th^* sequence of the *j^th^* and $length\left(Seq_{1}^{j}\right)\neq length\left(Seq_{2}^{j}\right)\ldots \neq length\left(Seq_{n}^{j}\right)$. The expansion form of a sequence $Seq_{i}^{\left(j\right)}$ is defend as $Seq_{i}^{\left(j\right)}=Seq_{i1}^{\left(j\right)},Seq_{i2}^{\left(j\right)},\ldots ,Seq_{il_{i}}^{\left(j\right)}$, where $Seq_{ik_{i}}^{\left(j\right)}\in \Sigma$. A set of sequences *S(j)* is either a heterogeneous sequence-set or a homogeneous sequence-set.

As we mentioned earlier in this paper, the data points under consideration are heterogeneous sequence-sets (e.g., genomes of viruses). For example, the genome of influenza virus is a segmented genome. The influenza genome has eight segments, each segment encoded into either 1 or 2 proteins [5,6,18-20]. Each protein has a biological function, and implicitly it has a nucleotide composition. The encoded proteins have different biological functions and different nucleotide compositions.

Without loss of generality, $\mathrm{let}\;\Xi \;=\;\{S^{\left(1\right)},\;S^{\left(2\right)},\;S^{\left(3\right)},\;\ldots \;,S^{\left(u\right)}\}$ be a collection of heterogeneous sequence-sets. Let *X* be a (*p*×1) feature vector (i.e., *X* ∈ R^*p*^). The feature vector *X* is a function from a data space to a feature space, *X*:Σ* - ∈ → R^*p*^. Let Ω = {ω_1_, ω_2_, …, ω_p_} be a set of strings (i.e., words, n-grams as defined in Cohen [21]), where ω_l_ ∈ (Σ* - *∈*)(*l*=1,2,…,p), and ∈ is the empty string. Let X_1_,X_2_,…,X_p_ be the features that constitute the feature vector *X*, where *X_i_* represents the number of occurrences of the string ω_l_ in a sequence Seq_i_^(j)^ ∈ S^(j)^, where S^(j)^ ⊂ (Σ* - ∈). Using the extension principle of data projection, define a transformation function *ϕ*(X): R^*p*^ → R^p'^, such that p'≥p. A transformation function *ϕ*(X) can be defined either as (1) a linear function, or as (2) a nonlinear function.

A transformation function is a mapping from one feature space (i.e., R^*p*^) to another feature space (i.e., R^p'^). It is either a linear or a nonlinear function. A feature space associated with a linear transformation function is called a linear feature space. A feature space associated with a nonlinear transformation function is called a nonlinear feature space. Using the extension principle of data projection, we have to extend the adaptability and applicability of the distance measure and the algorithms proposed in Daoud et al.’s studies [5,8]. A feature vector is a random vector. A function of feature vector is a feature vector. Suppose that *ϕ(X)* has the mean μ= E*ϕ(X)*= 0 and variance-covariance matrix ψ(*ϕ(X*))=Eϕ(X)ϕ(X)'. ψ(*ϕ(X*)): R^*p*^ → R^p'×p'^ is another mapping from R^*p*^ space to R^p'×p'^ space. After mapping sequence-sets form data space to feature space R^p'×p'^ using the composite transformation function ψ(*ϕ(X)*), and to proceed further, we have to extend the theoretical derivations of *D_ij_* (*γ*_1_) in any linear and nonlinear feature spaces.

#### Definition 3

Using principle (*P*_1_), mapping any heterogeneous sequence-set ((*S^(j)^* ⊂(Σ* - {∈})), where *S^(j)^* ∈ Ξ) into the feature space R^*p*^ can be achieved by mapping every sequence *Seq_i_^(j)^ ∈ S^(j)^* into the feature space R^*p*^ using a well defined (*p*×1) feature vector *X*. Hence, we result with a set of real-valued vectors ${\left\{X\left(i\right)\right\}}_{i=1}^{\left|S(j)\right|}$. Using principle (*P*_1_), mapping any heterogeneous sequence-set ((*S^(j)^* ⊂(Σ* - {∈})), where *S(j)* ∈ Ξ) into the feature space R^*p*^ can be achieved by mapping every sequence *Seq_i_^(j)^ ∈ S^(j)^* into the feature space R^*p*^ using a well defined (*p*×1) transformation function *ϕ(X)*. Hence, we result with a set of feature vectors ${\left\{\phi \left(X^{\left(i\right)}\right)\right\}}_{i=1}^{\left|S(j)\right|}$. Using principle (*P*_1_), mapping any heterogeneous sequence-set (*S^(j)^* ⊂(Σ* - {∈})), where *S^(j)^* ∈ Ξ) into the feature space R^p'×p'^ can be achieved by mapping ${\left\{\phi \left(X^{\left(i\right)}\right)\right\}}_{i=1}^{\left|S(j)\right|}$ into the feature space R^p'×p'^ using the (*p×p*) composite mapping $\psi \left(\left(\phi \left(X^{\left(i\right)}\right)\right)\right)$.

The following theorems represent the extension of theoretical derivations of the metric *D_ij_* (*γ*_1_) proposed in Daoud et al.’s studies [5,8] in any linear and nonlinear feature spaces, and consequently to justify the extension of the sequence-set analysis in any linear and nonlinear feature spaces. We use the generic derivation method used to obtain GPCA model (proposed by Flury in 1983 [14]) in the following theorem. It should be noted that GPCA is a statistical and computational generalized model of PCA, whereas the metric *D_ij_* (*γ*_1_) is a generalized metric.

##### Theorem 1

The distance between two heterogeneous sequence-set *S^(j1)^* and *S(^j2^)*, where *S^(j1)^* and *S^(j2)^* ∈ Ξ, is defined by the maximum deviation in variation between $\psi \left(\phi \left(X^{\left(j_{1}\right)}\right)\right)$ and $\psi \left(\phi \left(X^{\left(j_{2}\right)}\right)\right)$ embedded in the feature space R^p'×p'^.

*Proof:* The extended distance measure δ is a mapping from R^p'×p'^×R^p'×p'^→R^+^. Hence, let κ be a non-trivial vector in R^p'^. Define the linear combination G=κ'(ϕ(X^(j_1_)^-ϕ(X^(j_2_)^)). The required distance is defined in terms of *maximum deviation in variation*.

(2) max Var [G]

*subject to:* *κ* ∈ R*^p'^* *and norm(κ)*=1. Since *ϕ(X^(j_1_)^)* and *ϕ(X^(j_2_)^)* are statistically independent, we have:

(3) $$Var[G]\;=\;Var[\kappa '(\phi (X^{\left(j_{{}_{1}}\right)})-\phi (X^{\left(j_{2}\right)}))]$$

(4) $$\mathrm{Var}[G]\;=\;\mathrm{Var}[\kappa '(\phi (X^{\left(j_{{}_{1}}\right)})]-\mathrm{Var}[\kappa '\phi (X^{\left(j_{2}\right)}))]$$

(5) $$\mathrm{Var}[G]\;=\;E\;[\kappa '\phi (X^{\left(j_{1}\right)})\phi (X^{\left(j_{1}\right)})'\kappa ]\;-E[\kappa '\phi (X^{\left(j_{2}\right)})\phi (X^{\left(j_{2}\right)})'\kappa ]$$

(6) $$Var[G]\;=\kappa '[E\phi (X^{\left(j_{1}\right)})\phi (X^{\left(j_{1}\right)})'-E\phi (X^{\left(j_{2}\right)})\phi (X^{\left(j_{2}\right)})']\kappa$$

(7) $$Var[G]\;=\kappa '[\psi \left(\phi \left(X^{\left(j_{1}\right)}\right)\right)-\psi \left(\phi \left(X^{\left(j_{2}\right)}\right)\right)]\kappa$$

Maximizing Var[G] can be achieved by finding κ in R*^p'^* such that *norm(κ)*is equal to one. Let $\left|\alpha_{1}\right|<\left|\alpha_{2}\right|<\left|\alpha_{3}\right|<\left|\alpha_{4}\right|<\cdots <\left|\alpha_{\left(p'-1\right)}\right|<\left|\alpha_{\left(p'\right)}\right|$ be the ordered generalized eigenvalues associated with the generalized eigenvectors *κ_1_,κ_2_,κ_3_,κ_4_,…,κ_(p'-1)_,κ_(p')_* of the matrix [Ψ(*ϕ*(X^(J_1_)^)-Ψ(*ϕ*(X^(J_2_)^))] respectively. The maximum deviation in variation between Ψ(*ϕ*(*X^(j_1_)^*)) and Ψ(*ϕ*(*X^(j_2_)^*)) is given by the largest generalized eigenvalue $\left|\alpha_{1}\right|$ associated with the generalized eigenvector κ_1_. Hence, the generalized distance δ is defined by:

(8) $$\delta (\Psi (\phi (X^{\left(j_{1}\right)})),\Psi (\phi (X^{\left(j_{2}\right)})))=|\kappa_{1}^{'}[\Psi (\phi (X^{\left(J_{1}\right)}))-\Psi (\phi (X^{\left(J_{2}\right)}))]\kappa_{1}|$$

We used the key word *generalized* to differentiate the extension of *D_ij_* (*γ*_1_)in any linear and nonlinear feature spaces from the basic linear feature space associated with the basic transformation function *ϕ(X)=(X_1_,X_2_,…,X_p_)'*. Using the extension principle of data projection, the following theorem shows that the proposed generalized distance measure δ is a metric. The theorem is a generalization to the theorem given in Daoud’s study [5].

##### Theorem 2

The generalized distance measure δ(Ψ(ϕ(X^(j_1_)^)),Ψ(ϕ(X^(j_2_)^))): R^p'×p'^×R^p'×p'^ → R+ is a metric.

*Proof:* To show that *δ*(Ψ(*ϕ*(X^(j_1_)^)), Ψ(*ϕ*(X^(j_2_)^))) is a metric, *δ*(Ψ(*ϕ*(X^(j_1_)^)), Ψ(*ϕ*(X^(j_2_)^))) must satisfies the following properties.

(1) Reflexive: For any heterogeneous sequence-set $S^{\left(j_{1}\right)}\in \Xi ,\;\delta (\Psi (\phi (X^{\left(j_{{}_{1}}\right)})),\;\Psi (\phi (X^{\left(j_{{}_{2}}\right)})))=0\;\mathrm{iff}\;|\kappa_{1}^{'}[\Psi (\phi (X^{\left(j_{1}\right)}))-\Psi (\phi (X^{\left(J_{{}_{2}}\right)}))]\kappa_{1}|=0\;\mathrm{iff}\;[\Psi (\phi (X^{\left(j_{1}\right)}))=\Psi (\phi (X^{\left(j_{{}_{2}}\right)}))]$ (since κ_1_ is a non-trivial vector embedded in R^p'^).

(2) Symmetric: For any two heterogeneous sequence-sets *S*^(j_1_)^ and *S*^(j_2_)^ ∈ Ξ, $\delta (\Psi (\phi (X^{\left(j_{1}\right)})),\;\Psi (\phi (X^{\left(j_{2}\right)})))=|\kappa_{1}^{'}[\Psi (\phi (X^{\left(J_{1}\right)}))-\Psi (\phi (X^{\left(J_{2}\right)}))]\kappa_{1}|=|(-1)\kappa_{1}^{'}[\Psi (\phi (X^{\left(J_{2}\right)}))-\Psi (\phi (X^{\left(J_{1}\right)}))]\kappa_{1}|\;=|(-1)||\kappa_{1}^{'}[\Psi (\phi (X^{\left(J_{2}\right)}))-\Psi (\phi (X^{\left(J_{1}\right)}))]\kappa_{1}|=|\kappa_{1}^{'}[\Psi (\phi (X^{\left(J_{2}\right)}))-\Psi (\phi (X^{\left(J_{1}\right)}))]\kappa_{1}|=\delta (\Psi (\phi (X^{\left(j_{2}\right)})),\;\Psi (\phi (X^{\left(j_{1}\right)})))$.

(3) Positive: For any two heterogeneous sequence-sets *S*^(j_1_)^ and *S*^(j_2_)^ ∈ Ξ, $\delta (\Psi (\phi (X^{\left(J_{1}\right)})),\;\Psi (\phi (X^{\left(j_{2}\right)})))\;=\;|\kappa_{1}^{'}[\Psi (\phi (X^{\left(J_{1}\right)}))-\Psi (\phi (X^{\left(j_{2}\right)}))]\kappa_{1}|=|\alpha_{1}|\geq 0$, where α_1_ ∈ R.

(4) Transitive: For any heterogeneous sequence-sets *S^(j_1_)^*,*S*^(j_2_)^, and *S*^(j_3_)^ ∈ Ξ, δ(Ψ(*ϕ*(X^(j_1_)^)), Ψ(*ϕ*(X(^(j_2_)^)))=|κ'_1_ [Ψ(*ϕ*(X^(j_1_)^))-Ψ(*ϕ*(X(^(j_2_)^))] κ_1_ |= |κ'_1_ [Ψ(ϕ(X^(j_1_)^))- Ψ(*ϕ*(X(^(j_2_)^)) + Ψ(*ϕ*(X(^(j_3_)^)) - Ψ(ϕ(X(^(j_3_)^))] κ_1_ |= |κ'_1_ [[Ψ(*ϕ*(X(^(j_1_)^) )-Ψ(*ϕ*(X(^(j_3_)^))]+[(Ψ(*ϕ*(X(^(j_3_)^))-Ψ(*ϕ*(X(^(j_2_)^))]] κ_1_ |= |[κ'_1_ [Ψ(*ϕ*(X^(j_1_)^))-Ψ(*ϕ*(X(^(j_3_)^)) κ_1_]+[κ'_1_ Ψ(*ϕ*(X(^(j_3_)^))-Ψ(*ϕ*(X(^(j_2_)^))κ_1_]]|≤|κ'_1_ [Ψ(*ϕ*(X(^(j_1_)^)-Ψ(*ϕ*(X(^(j_3_)^))] κ_1_ |+|κ'_1_ [Ψ(*ϕ*(X(^(j_3_)^))- Ψ(*ϕ*(X(^(j_2_)^))] κ_1_ |=δ(Ψ(*ϕ*(X^(j_1_)^)),Ψ(*ϕ*(X(^(j_3_)^)))+δ(Ψ(*ϕ*(X(^(j_3_)^ )),Ψ(*ϕ*(X(^(j_2_)^)))

The proposed generalized metric δ can be used in performing classification and clustering tasks on heterogeneous sequence-sets in any linear and nonlinear feature spaces. The efficiency and sufficiency of using the mean of the distance values of δ instead of using the minimum or the median of the distance values in solving the classification problem of heterogeneous sequence-sets in any linear and nonlinear feature spaces are presented below. The following theorem assumes that the proposed metric with various transformation functions and data sets is a random variable with unknown distribution. Therefore, a random sample of the proposed metric should be considered.

##### Theorem 3

Given classes of heterogeneous sequence-sets Ξ_1_,Ξ_2_,…,Ξ_k_ (labeled datasets). Given an unlabeled query sequence-set QSS. The label of the given query sequence-set QSS is defined by: label(QSS)=argmini{δΞ1}i=1k, δΞi=Ξi-1∑j=1Ξi□δ(ψ(ϕ(X(j))), ψ(ϕ(X(QSS)))

which is the best classification decision compared with the classification decisions: $label(QSS)=argmin_{i}{\left\{min(\Xi i)\right\}}_{i=1}^{k}$, where $\min(\Xi_{i})=min{\left\{\delta (\Psi (\phi (X\left(j\right))),\;\Psi (\phi (X\left(QSS\right))))\right\}}_{j=1}^{\left|\Xi_{i}\right|}$ and $label(QSS)={\mathrm{argmin}}_{i}\{{\mathrm{median\Xi}}_{i}{\}}_{i=1}^{k}$, where ${\mathrm{median\Xi}}_{i}=median{\left\{\delta (\Psi (\phi (X\left(j\right))),\;\Psi (\phi (X\left(\mathrm{QSS}\right))))\right\}}_{j=1}^{\left|\Xi_{i}\right|}$

In this context, |Ξ_i_| represents the number of sequence-sets in Ξ_i_.

##### Proof

∀i, measuring the distance between the unlabeled query sequence-set S^(QSS)^ with every using the proposed generalized metric δ(,), we result with a sample of distance values δ_i1_,δ_i2_,…, δ_i|Ξ_i_|_, where δ_ij_=δ(Ψ(ϕ(X^(j)^)), Ψ(ϕ(X^(QSS)^))). In addition, $\delta_{i(1)},\delta_{i(2)},\ldots ,\delta_{{}_{i}(|\Xi_{i}|)}$ represent the ordered sample of $\delta_{i1},\delta_{i2},\ldots ,\delta_{{}_{i}(|\Xi_{i}|)}$. Without loss of generality, suppose that the mean and the variance of the *ith* sample are denoted by *ξ_i_* and *ϑ_i_* respectively (i.e., *E[δ_i_] = ξ_i_ and* Var*[δ_i_]=ϑ_i_)*. Let *min_Ξi_=δ_i(1)_* be the minimum of the *ith* sample. Let $median_{\Xi_{i}}=\delta_{i\left(\frac{\left|\Xi_{i}\right|+1}{2}\right)}$ if $\left|\Xi_{i}\right|$ is odd, otherwise let $median_{\Xi i}\;=\frac{1}{2}\left(\delta_{{}_{i}\left(\frac{\left|\Xi_{i}\right|}{2}\right)}+\delta_{{}_{i}\left(\frac{\left|\Xi_{i}\right|+2}{2}\right)}\right)$ if $\left|\Xi_{i}\right|$ is even. Let δΞi be the mean of the *ith* sample. $E[\delta_{i(1)}]=\xi_{i},\;E[\delta_{i\left(\frac{|\Xi_{i}|+1}{2}\right)}]=\xi_{i}\;if|\Xi_{i}|$ *is odd,* $E\frac{1}{2}\left(\delta_{i\left(\frac{\left|\Xi_{i}\right|}{2}\right)}+\delta_{i\left(\frac{|\Xi_{i}|+2}{2}\right)}\right)=\frac{1}{2}E[\delta_{i\left(\frac{\left|\Xi_{i}\right|}{2}\right)}]+\frac{1}{2}E[\delta_{i\left(\frac{|\Xi_{i}|+2}{2}\right)}]=\frac{1}{2}\xi_{i}+\frac{1}{2}\xi_{i}=\xi_{i}if|\Xi_{i}|$ *is even,* and E[δΞi]=ξi. Var[δi(1)]=ϑi,Var[δi|Ξi|2]=ϑiif|Ξi| is odd, $Var\frac{1}{2}(\delta_{i\left(\frac{\left|\Xi_{i}\right|}{2}\right)}+\delta_{i\left(\frac{|\Xi_{i}|+2}{2}\right)})=\frac{1}{2}\vartheta_{i}if|\Xi_{i}|$ is even, and Var[δΞi)]=|Ξi|-1)ϑi. Hence, Var[δΞi]<Var[δi(1)]=Var[δi|Ξi|+12] (if $\left|\Xi_{i}\right|$ is odd), and Var[δΞi]<Var12(δi(|Ξi|2)+δi(|Ξi|+22))<Var[δi(1)](if|Ξi| is even). Thus, δΞi is the best parameter that can be used in a classification decision rule to classify composite data points under consideration.

Moreover, based on the consistency definition given in Hogg and Craig [22] and using *Chebyshev'*s inequality, the consistency of the proposed decisions can be evaluated as follows:

(9) $$Pr(|\theta_{min}-min_{\Xi_{i}}<\rho |)\geq 1-\frac{\vartheta_{i}}{\rho^{2}}$$

and

(10) $$Pr(|\theta_{median}-median_{\Xi_{i}}<\rho |)\geq 1-\frac{\vartheta_{i}}{\rho^{2}}$$

and

(11) Pr(|θδ-δΞi<ρ|)≥1-ϑi|Ξi|ρ2

Now: $lim_{|\Xi_{i}|\to \infty }(1-\frac{\vartheta_{i}}{\rho^{2}})\neq 1\;while\;lim_{|\Xi_{i}|\to \infty }(1-\frac{\vartheta_{i}}{|\Xi_{i}|\rho^{2}})=1,\;where\;\rho >0$. 

#### Definition 4 (classification)

Let QSS be a query sequence-set. Let Ξ_1_,Ξ_2_,…,Ξ_k_ be k classes of heterogeneous sequence-sets. Let ${\left\{\delta \mathrm{ij}\right\}}_{j=1}^{\left|\mathrm{\Xi i}\right|}$ be the distance values results from comparing the QSS with each sequence-set in Ξ_i_. Without loss of generality, suppose that ${\left\{\delta \mathrm{ij}\right\}}_{j=1}^{\left|\mathrm{\Xi i}\right|}$ has the normal distribution with mean ξ_i_ and variance ϑ_i_. Hence, the sample mean of the generalized distance metric δΞi is a sufficient statistic for ξ_i_.

The proof of the sufficiency condition of the sample mean of the normal distribution is straightforward [22] and it can be used to prove that the sample mean of the generalized distance metric δΞi is a sufficient statistic for ξ_i_.The joint probability density function of ${\left\{\delta \mathrm{ij}\right\}}_{j=1}^{\left|\mathrm{\Xi i}\right|}$ can be written as:

(12) $$f(\delta_{i1},\delta_{i2},\delta_{i3},\ldots ,\delta_{{}_{i}|\Xi_{i}|};\xi_{i})=f(\delta_{i1};\xi_{i})\times f(\delta_{i2};\xi_{i})\times f(\delta_{i3};\xi_{i})\times \ldots f(\delta_{{}_{i}|\Xi_{i}|};\xi_{i})$$

(13) $$f(\delta_{i1};\xi_{i})\times f(\delta_{i2};\xi_{i})\times f(\delta_{i3};\xi_{i})\times \ldots f(\delta_{i}|\Xi_{i}|;\xi_{i})={\frac{1}{\sqrt{2\pi }\vartheta_{i}}}^{\left|\Xi_{i}\right|}e^{-\frac{\sum_{j=1}^{\left|\Xi_{i}\right|}{\left(\delta_{ij}-\zeta_{i}\right)}^{2}}{2\zeta_{i}^{2}}}$$

(14) f(δi1;ξi)×f(δi2;ξi)×f(δi3;ξi)×…f(δi|Ξi|;ξi)=12πϑiΞie-∑j=1Ξiδij-δΞi-ξi-δΞi2/2ϑi2

(15) f(δi1;ξi)×f(δi2;ξi)×f(δi3;ξi)×…f(δi|Ξi|;ξi)=12πϑiΞie-∑j=1Ξiδij-δΞi22ϑ2e-Ξiζi-δΞi22ϑ2

We just factorized the joint probability density function of ${\left\{\delta ij\right\}}_{j=1}^{\left|\Xi_{i}\right|}$ into two factors. The first factor depends upon ${\left\{\delta ij\right\}}_{j=1}^{\left|\Xi_{i}\right|}$, while the second factor depends upon $\xi_{i}\;and\;{\left\{\delta ij\right\}}_{j=1}^{\left|\Xi_{i}\right|}$. Hence, under the assumption that ϑ_i_ is known, we conclude that δ is a sufficient statistic for ξ_i_ (Sufficiency theorem [22]). In accordance with the proposed extension principle of projecting composite data points into various linear or nonlinear feature spaces, we use the generalized metric δ, instead of *D_ij_* (*γ*_1_), to modify the classification and clustering algorithms proposed in Daoud’s study [5]. The adapted algorithms can be used in classifying and clustering composite data points in any linear and nonlinear feature spaces.

In the next part of this section, we present the necessary and sufficient condition for generating a heterogeneous sequence-set from a real heterogeneous sequence-set (i.e., real composite data point) by using simulation.

#### Definition 5

Let Σ be a finite alphabet. Let Ω={ω_1_,ω_2_,…,ω_p_ } be a set of strings, where ω_l_∈(Σ*-ϵ)(*l*=1,2,…,*p*),*and ϵ* is the empty string. Let *S^(j)^={Seq_1_^(j)^,Seq_2_^(j)^,…,Seq_n_^(j)^}* be a set of heterogeneous sequences, where *Seq_i_^(j)^* is the ith sequence of the *jth* sequence-set. Let *X=(X_1_,X_2_,X_3_,…,X_p_)'* be a (*p×1*) feature vector embedded in R^*p*^, where X_l_ represents the occurrences of ω_1_∈ (*l*=1,2,…,*p*). A necessary and sufficient condition for generating a heterogeneous sequence-set $\hat{S^{J}}=\{\hat{S}e{q_{1}}^{\left(j\right)},\;\hat{S}e{q_{2}}^{\left(j\right)},\;\ldots ,\;\hat{S}e{q_{n}}^{\left(j\right)}\}$ from a real sequence-set S^(j)^ is:∀*i*, $F_{X,n'}^{\left(i\right)}$, (x)converges in distribution to *F_X_(x)* as *n'*→ ∞, where $F_{X}^{\left(i\right)}$ *(x)* is the distribution function of the nucleotide composition of $Seq_{i}^{\left(j\right)}$, and *n'* ∈ R is proportionally related to the length of $Seq_{i}^{\left(j\right)}$.

In definition (5), we presented the necessary and sufficient condition for generating a heterogeneous sequence-set from a real heterogeneous sequence-set by using simulation. The robust sequence-set generator is built upon using built-in matlab functions, and it has the following computational steps: the nucleotide composition of each sequence in a given real heterogeneous sequence-set is estimated to generate a simulated sequence with longer length, and hence, to compose a simulated heterogeneous sequence-set.

We remark the following: A transformation function is a function of random feature vectors. It is a measurable function. A function of random feature vectors is a random feature vector. Therefore, a transformation function is measurable and parametric-free (i.e., statistic). Hence, we aim to observe the effect of linear and non-linear transformation functions on the classification and clustering results using *D_ij_* (*γ*_1_). In this context, the formulation of any transformation function is based on the following: (1) linearity or non-linearity of the random feature vectors, and (2) use of special functions of random feature vectors, for example, first

order statistic, last order statistic, and standard deviation. Different transformation functions can be used to map sequence-sets into different feature spaces. Composite transformation is another alternative for mapping sequence-sets. In this paper, we aim to compare linear vs no-linear transformation functions that can be used in mapping composite data points (i.e., heterogeneous sequence-sets) into feature spaces.

In this section, we presented the extended theory of the proposed largest generalized eigenvalue based distance metric *D_ij_* (*γ*_1_) in arbitrary feature spaces. In addition, we presented the theoretical properties of *D_ij_* (*γ*_1_) in arbitrary feature spaces as a metric, and the efficiency of using the decision rule δΞi in supervised classification compared with the decision rules *min_Ξ__i_* and *median_Ξ__i_*. Moreover, we presented the sufficiency of using the decision rule δΞi in supervised classification of heterogeneous sequence-sets. In the next section, experiments and results are presented.

## Results

The experiments and results are presented in this section. We perform two experiments to analyze heterogeneous sequence-sets in linear and nonlinear feature spaces. The objective of the first experiment is as follows: we focus on graphically analyzing patterns (clusters, dispersion maps of clusters, limiting dispersion maps of clusters) in real heterogeneous sequence-sets, whereas, the objective of the second experiment is as follows: we focus on testing the effect of the lengths of sequences in sequence-sets generated by simulation on classification and clustering results.

### The first experiment: analyzing real heterogeneous sequence-sets

**Fig. 4.**
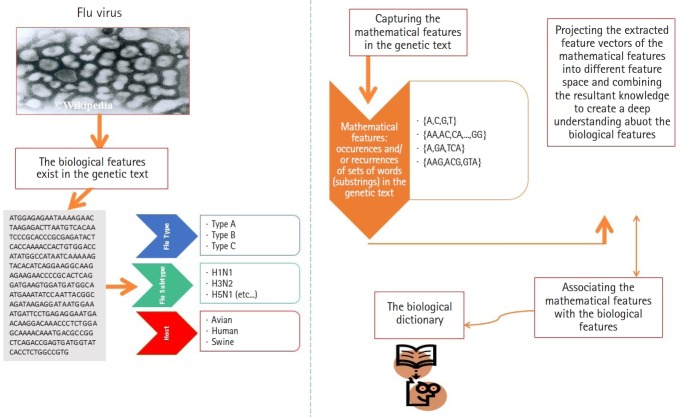
Analyzing the segmented genome of inﬂuenza virus.

In this subsection, we present the first experiment. In the first experiment, we focus on analyzing real heterogeneous sequence-sets in linear and non-linear feature spaces. The heterogeneous sequence-sets under consideration are segmented genomes of the influenza virus. The genome has eight segments, each segment encoded into one or two proteins. The encoded proteins have different biological functions. The segmented genome of influenza virus has highly mutation rates. Therefore, the influenza virus has negative impacts on the public health. The main biological features of the influenza virus are (1) virus type, (2) virus subtype, and (3) hosts. The main types of the influenza virus are A, B, and C. The influenza A-virus has various subtypes, for example, H1N1, H2N1, H3N2, and H5N1. The subtype variations are embedded in the surface proteins of influenza genome. The main hosts of the influenza virus are avian, human, and swine. The main biological features of the influenza virus are expected to be hidden in the genetic text of the influenza genome (Fig. 4). Each biological feature is expected to be represented by one or more hidden information structures in the genetic text of the influenza genome. Therefore, mining the genetic text of the influenza virus is the key point in analyzing the biological features of the influenza genome. To be consistent with the scope and objectives of this paper, we present only the useful biological details of the influenza genome [19,20,23-25]. We downloaded real datasets from NCBI's Influenza Virus Sequence Database [26]. The real datasets are the segmented genomes of the influenza virus (real heterogeneous sequence-sets). We downloaded 30 segmented genome of the influenza virus (type: A, subtype: H1N1, host: assorted, geographical areas: assorted) to represent class Ξ_1_, 30 segmented genome of the influenza virus (type: B, host: human, geographical areas: assorted) to represent class Ξ_2_, and 45 segmented genome of the influenza virus (type: A, B , subtype of A: H1N1, host: assorted, geographical areas: assorted) to represent unlabeled heterogeneous sequence-sets *UnLabeled*. We use Ξ_1_, Ξ_2_, and *UnLabeled* to perform classification experiments in linear and nonlinear feature spaces using δ. We combine Ξ_1_ and Ξ_2_ (Ξ. = Ξ_1_ ∪ Ξ_2_, $\left|\Xi \right|=60$ sequence-set) to perform clustering experiments in linear and nonlinear feature spaces using δ ®.

To be consistent with research objective of this paper (see the research statement), we formulate the transformation functions as either linear or nonlinear transformation functions(arbitrary). There is no restriction on how to define a transformation function(user-defined), but certainly it depends on the complexity of composite data points, and the type and the quality of targeted information in composite data points under consideration (e.g., to minimize the classification errors). We perform the classification and clustering experiments using two transformation functions in order to show the impact of extracted information from datasets under consideration on the classification and clustering results in linear and nonlinear feature spaces, and for illustration purposes, the classification and clustering results are projected into a two-dimensional space.

**Fig. 5.**
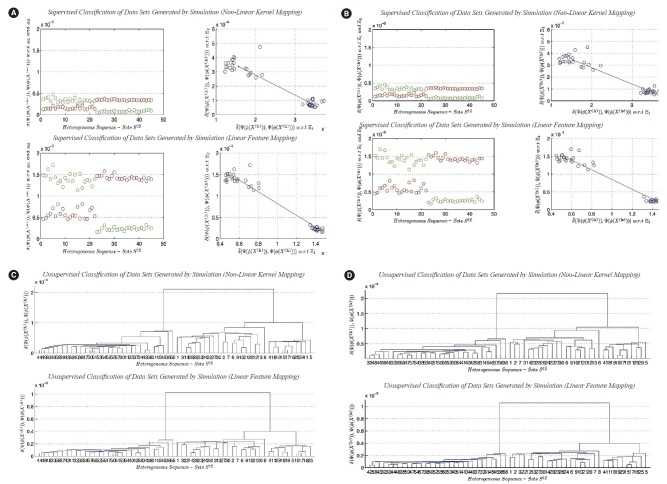
(A) Classiﬁcation of heterogeneous sequence-sets (real data sets): (1) non-linear transformation function (*ϕ*_2_ *(X)*), and (2) linear transformation function (*ϕ*_1_ *(X)*). (B) Classiﬁcation of heterogeneous sequence-sets generated by simulation identical to real data sets (sequence length, ×200): (1) non-linear transformation function (*ϕ*_2_ *(X)*), and (2) linear transformation function (*ϕ*_1_ *(X)*). (C) Clustering of heterogeneous sequence-sets (real data sets): (1) non-linear transformation function (*ϕ*_2_ *(X)*), and (2) linear transformation function (*ϕ*_1_ *(X)*). (D) Clustering of heterogeneous sequence-sets generated by simulation identical to real data sets (sequence length, ×200): (1) non-linear transformation function (*ϕ*_2_ *(X)*), and (2) linear transformation function (*ϕ*_1_ *(X)*).

**Fig. 6.**
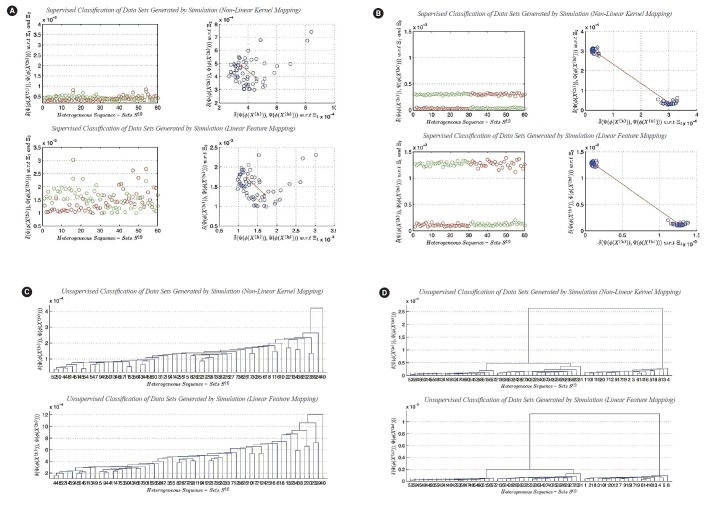
(A) Classiﬁcation of heterogeneous sequence-sets generated by simulation (sequence length, ×1): (1) non-linear transformation function (*ϕ*_2_ *(X)*), and (2) linear transformation function (*ϕ*_1_ *(X)*). (B) Clustering of heterogeneous sequence-sets generated by simulation (sequence length, ×1): (1) non-linear transformation function (*ϕ*_2_ *(X)*), and (2) linear transformation function (*ϕ*_1_ *(X)*). (C) Classiﬁcation of heterogeneous sequence-sets generated by simulation (sequence length,×100): (1) nonlinear transformation function (*ϕ*_2_ *(X)*), and (2) linear transformation function (*ϕ*_1_ *(X)*). (D) Clustering of heterogeneous sequence-sets generated by simulation (sequence length, ×100): (1) non-linear transformation function (*ϕ*_2_ *(X)*), and (2) linear transformation function (*ϕ*_1_ *(X)*).

The results are collected and presented in Figs. 5 and 6. To distinguish the research work presented in this paper from the research work presented in Daoud’s study [5], we focus on graphically analyzing patterns in the datasets under consideration from the following angles: (1) the exact and limiting dispersion maps of each cluster, and (2) the distance between clusters (i.e., margins between clusters). In other words, we aim to graphically analyze patterns in datasets embedded in high dimensional linear and non-linear feature spaces without using classical evaluation measures that are usually used in evaluating classification and clustering results (i.e., to approximately deduce the empirical conclusions directly from results). The empirical conclusions approach is another approach that can be used in X-raying and analyzing the existing patterns in datasets under consideration using the following (empirical analysis based on deterministic parameters): the Euclidean distance between clusters, the exact dispersion map, the limiting dispersion map, the expected number of clusters (and/or sub-clusters), and the observed number of clusters (and/or sub-clusters). It is away from assuming

mathematical statistics assumptions to make mathematical statistics decisions on clustering and classification results. In this paper, the four feature variables *X*_1_, *X*_2_, *X*_3_, and *X*_4_ represent the occurrences of the possible four 1-grams in each bio-sequence in a sequence-set respectively. The feature extraction technique used in the experiments is the n-gram technique. The n-gram feature extraction technique is well known technique in natural language processing. The feature extraction mechanism is given in definition 3. In the future work, we aim to analyze the occurrences of 2-grams and 3-grams in each bio-sequence in a sequence-set using linear and non-linear transformation functions (the results are not shown in this paper due to space limitations).

To answer the research questions proposed in this paper, we use two experimental approaches to reach the empirical conclusions on analyzing heterogeneous sequence-sets in linear and non-linear feature spaces. In the first approach, we use the real datasets that are previously defined in this section to perform the classification and clustering experiments. In the second approach, we use datasets generated by simulation using the empirical distributions of the nucleotide compositions of the real datasets that are previously defined in this section to perform the classification and clustering experiments. The generated datasets have the following characteristic: the sequence length of each sequence in a generated sequence-set is multiplied by the factor 200 in order to reach a good approximation to the exact distribution of nucleotide composition (Limiting Distribution). For each transformation function, the empirical pattern analysis of classification and clustering results is given below.

$Comparing\;\phi_{1}(X)=(X_{1},\;X_{2},\;\ldots ,\;X_{p}{)}^{'}\;versus\;\phi_{2}(X)=(X_{1}^{2},\;X_{2}^{2},\;\ldots ,\;X_{p}^{2}{)}^{'}$

In this subsection, we present a comparison between the two transformation functions *ϕ*_2_*(X)* (non-linear function, quadratic in all feature-axises) and *ϕ*_1_*(X)* (Linear function). Both functions have the dimensionality: p=4. The classification results of *ϕ*_1_*(X)* versus *ϕ*_2_*(X)* are given in Fig. 5A and 5B, respectively. Fig. 5A represents the classification results using real datasets in classes Ξ_1_, Ξ_2_, and *UnLabeled*. Fig. 5B represents the classification results of datasets generated by simulation using the limiting distributions of the nucleotide compositions of real datasets in classes Ξ_1_, Ξ_2_, and *UnLabeled*. Each figure is consisting of four sub-figures. The upper left sub-figure represents the distance of each unlabeled composite data point in *UnLabeled* with respect to classes Ξ_1_, Ξ_2_ respectively using *ϕ*_1_*(X)* (inclusively, visualize the classification errors and the variation of each cluster). The upper right sub-figure represents the supervised classification result (scatter diagram) of the composite data points in *UnLabeled* using *ϕ*_1_*(X)*. The lower left sub-figure represents the distance of each unlabeled data point in *UnLabeled* with respect to classes Ξ_1_, Ξ_2_ respectively using *ϕ*_2_*(X)* (inclusively, visualize the classification errors and the variation of each cluster). The lower right sub-figure represents the supervised classification result (scatter diagram) of the composite data points in *UnLabeled* using *ϕ*_2_*(X)*. Both figures show the existence of two main clusters, one with high variations (upper-left) and the other with low variation (right-lower). Let c_1_ be the cluster with high variations and let c_2_ be the cluster with low variations. Both figures illustrate the following empirical conclusion: the distance between c_1_ and c_2_ in non-linear feature space *ϕ*_2_*(X)* is less than the distance between c_1_ and c_2_ in linear feature space *ϕ*_1_*(X)*. The clusters c_1_ and c_2_ are well-separated. The dispersion map of c_1_ indicates the existence of sub-clusters. In other words, few composite data points are located with significant deviation in variations from the centroid of c_1_. This empirical conclusion remains unchanged (valid) in the case of using the limiting distribution. The question that arises in this context can be summarized as follows: what is the impact of the previous empirical conclusion on the biological-side? In this case and based on the dispersion maps of c_1_ (in the case of the exact and limiting distributions), it is clear that the genome of type-A H1N1-flu virus has a high mutation rate and can be housed by various types of hosts. To proceed further in answering the question, for example, if the annual vaccine is designed by selecting a virus with a genome close to the centroid of c_1_, then in this case, the efficiency of the produced vaccine is expected to be affected with a percentage during the flu-season. In fact, it is probably the efficiency of the vaccine is expected to be reduced by a percentage, and consequently it has a significant impact on the public health. The dispersion map of c_1_ indicates the following: the virus may cause symptoms with high variations. The dispersion map of c_2_ indicates the compactness of the cluster. The dispersion map of c_2_ with respect to the limiting distribution shows a bit more variations among composite data points (i.e., sequence-sets) compared with the dispersion map of c_2_ with respect to the exact distribution of the nucleotide composition. Hence, type-B flu virus may causes symptoms with low variations, and therefore, if the annual vaccine is designed by selecting a virus with a genome close to the centroid of c_2_, then in this case, the efficiency of the produced vaccine is expected to be affected with a very small percentage during the flu-season. The clustering results of *ϕ*_1_*(X)* versus *ϕ*_2_*(X)* are given in Fig. 5C and 5D, respectively. It should be noted that some of the empirical conclusions deduced from the classification results can be deduced from the clustering results. For example, the main clusters c_1_ (right cluster, see Fig. 5C and 5D) and c_2_ (left cluster, Fig. 5C and 5D) are well-separated. The cluster c_1_ has sub-clusters, but we can not provide biological interpretations about the existence of sub-clusters due to lack of biological/medical information associated with the real data sets under consideration (i.e., segmented genome of flu-virus).

### The second experiment: analyzing simulated-based heterogeneous sequence-sets

In this subsection, we present the second experiment. In the second experiment, we focus on testing the effect of the lengths of sequences in sequence-sets generated by simulation on classification and clustering results in linear and non-linear feature spaces. In the real world, sometimes we face lack of data or information about a specific biological phenomenon. In order to overcome this obstacle, we generate datasets using simulation. Simulation is a well-known technique in the areas of statistical computing, performance modeling, and other research areas. We downloaded three types of segmented genomes of influenza virus from NCBI (Influenza Virus Resource) [26]. Those types are randomly selected: (1) Real-Dataset(1): influenza A virus (H1N1, Human, USA, 2011), (2) Real-Dataset(2): influenza B virus (Human, Thailand, 2012), (3) Real-Dataset(3): influenza C virus (Swine, USA, 2011). We use the three real datasets of influenza virus (i.e., three segmented genomes) to generate three random samples of segmented genomes using simulation. In other words, we generate composite data points by using only one real composite data point from each virus type as a prototype. We use the nucleotide compositions of sequences in each randomly selected prototype sequence-set to generate a sample of composite data points from each virus type. Each random sample has the size 20. Discovering the impact of the parameter *n'* given in (Definition 5) on the performance of the clustering task using the largest generalized eigenvalue-based distance metric *D_ij_* (*γ*_1_) in feature space can be achieved by the following design of experiment. We generate the three random samples at $n'=\left|Se{q_{i}}^{\left(j\right)}\right|,\;10\;\times \;\left|Se{q_{i}}^{\left(j\right)}\right|,\;20\;\times \;\left|Se{q_{i}}^{\left(j\right)}\right|,\;30\;\times \;\left|Se{q_{i}}^{\left(j\right)}\right|,\;40\;\times \;\left|Se{q_{i}}^{\left(j\right)}\right|,\;50\;\times \;\left|Se{q_{i}}^{\left(j\right)}\right|,\;\mathrm{and}\;100\;\left|Se{q_{i}}^{\left(j\right)}\right|,\;\mathrm{where}\;\left|Se{q_{i}}^{\left(j\right)}\right|$ represent the sequence length of $Se{q_{i}}^{\left(j\right)}$, ∀*i* and ∀*j*. The generated composite data points that are used in performing clustering experiment are different from the composite data points that are used in performing classification experiment. However, both collections of composite data points are generated using the same prototypes (i.e., same real composite data points). As we mentioned in this section, we aim to compare the impact of the sequence length generated by simulation on calcification and clustering results. There are three factors that may have an impact on the calcification and clustering results: (1) the selected feature vector (in this paper: *X* represents the occurrences of all-possible 1-grams), (2) the limiting distribution of the nucleotide composition (i.e.,*n'*), and (3) the transformation functions. In this paper, we present the worst case $\left(n'=\left|Se{q_{i}}^{\left(j\right)}\right|\right)$ and the best case $\left(n'=100\times \left|Se{q_{i}}^{\left(j\right)}\right|\right)$ for the calcification and clustering results using the proposed transformation functions due to space limitations. Those results are illustrated in Fig. 6A–6D.

#### The results of classification

In this subsection, we discuss the results of classification in linear and non-linear feature spaces (only two-classes classification problem considered). The best cases for the classification results indicate the existence of the two main clusters. The two main clusters are well separated. The distance between the two centroids is vary from one feature space to another. It depends upon the mathematical definition of transformation functions. In this subsection, we can not analyze the dispersion maps of each cluster since the composite data points used in this experiment are generated using one prototype of nucleotide compositions for each class of sequence-sets. The worst cases for the classification results indicate the impact of the sequence length n^' on supervised classification in linear and non-linear feature spaces. The worst cases empirically indicate the following: there is no sufficient scientific evidence support the existence of two different clusters in composite data points under consideration.

#### The results of clustering

In this subsection, we discuss the results of clustering in linear and non-linear feature spaces. In this experiment, the composite data points are generated by simulation, and they contain three main clusters. The best cases of clustering results indicate the existence of the three main clusters, whereas, the worst cases of clustering results devote the following empirical conclusion: there is no sufficient scientific evidence support the existence of the three different main clusters in composite data points under consideration.

In this section, we presented the classification and clustering results in linear and non-linear feature spaces. The experiments are performed using composite data points generated by simulated. Each composite data point represents a heterogeneous sequence-set. In the next section, we present conclusions and future work.

## Discussion

**Fig. 7.**
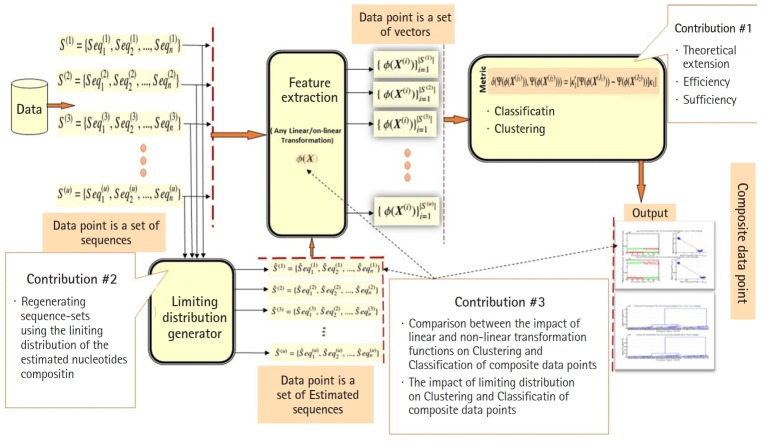
The extension of the proposed variance-covariance statistical pattern recognition system (Statistical Learning) in any linear and non-linear feature spaces with capability of using limiting distribution generator.

In this section, we present conclusions and future work. The main contributions of this paper can be summarized as follows (Fig. 7). We extended the theoretical-side of the largest generalized eigenvalue-based distance measure D_ij_ (*γ*_1_) in any linear and non-linear feature spaces. We proved that the proposed measure D_ij_ (*γ*_1_) in Daoud’ study [5,6,8] satisfies the properties of a metric space under any linear or non-linear transformation function. We proved the sufficiency and efficiency of using the decision rule δΞi (i.e., mean of D_ij_ (γ_1_)) in classification compared with the decision rules *min_Ξ__i_* and *median_Ξ__i_*. We showed the impact of the sequence-length n' used in generating composite data points on classification and clustering results in linear and non-linear feature spaces. We proposed two new main concepts in this context: the exact dispersion map and the limiting dispersion map of a cluster. The feature vector used in this paper represents the occurrence of all possible single nucleotides (i.e., 1-grams) in each sequence of a heterogeneous sequence-set. The variations of 1-grams have an important application in genetic evolution (Single Nucleotide Polymorphisms). In the future work, we aim to analyze the impact of using the occurrences of 2-grams and 3-grams in heterogeneous sequence-sets on classification results in any linear and non-linear feature spaces using various designs of experiments.
